# A Comparative Metagenomic Analysis of Specified Microorganisms in Groundwater for Non-Sterilized Pharmaceutical Products

**DOI:** 10.1007/s00284-024-03791-w

**Published:** 2024-07-17

**Authors:** Soumana Daddy Gaoh, Pierre Alusta, Yong-Jin Lee, John J. LiPuma, David Hussong, Bernard Marasa, Youngbeom Ahn

**Affiliations:** 1 Division of Microbiology, National Center for Toxicological Research, U.S. Food and Drug Administration, 3900 NCTR Road, Jefferson, AR 72079-9502 USA; 2 Division of Systems Biology, National Center for Toxicological Research, U.S. Food and Drug Administration, Jefferson, AR 72079 USA; 3 Department of Natural Sciences, Albany State University, Albany, GA 31707 USA; 4 Department of Pediatrics, University of Michigan, Ann Arbor, MI 48109 USA; 5 Eagle Analytical Services, Houston, TX 77099 USA; 6 Office of Pharmaceutical Quality, Center for Drug Evaluation and Research, U.S. Food and Drug Administration, Silver Spring, MD 20993 USA

## Abstract

In pharmaceutical manufacturing, ensuring product safety involves the detection and identification of microorganisms with human pathogenic potential, including *Burkholderia cepacia* complex (BCC), *Escherichia coli, Pseudomonas aeruginosa*, *Salmonella enterica, Staphylococcus aureus, Clostridium sporogenes, Candida albicans,* and *Mycoplasma* spp., some of which may be missed or not identified by traditional culture-dependent methods. In this study, we employed a metagenomic approach to detect these taxa, avoiding the limitations of conventional cultivation methods. We assessed the groundwater microbiome’s taxonomic and functional features from samples collected at two locations in the spring and summer. All datasets comprised 436–557 genera with *Proteobacteria, Bacteroidota, Firmicutes, Actinobacteria,* and *Cyanobacteria* accounting for > 95% of microbial DNA sequences. The aforementioned species constituted less than 18.3% of relative abundance. *Escherichia* and *Salmonella* were mainly detected in Hot Springs, relative to Jefferson, while *Clostridium* and *Pseudomonas* were mainly found in Jefferson relative to Hot Springs. Multidrug resistance efflux pumps and BlaR1 family regulatory sensor-transducer disambiguation dominated in Hot Springs and in Jefferson. These initial results provide insight into the detection of specified microorganisms and could constitute a framework for the establishment of comprehensive metagenomic analysis for the microbiological evaluation of pharmaceutical-grade water and other non-sterile pharmaceutical products, ensuring public safety.

**Supplementary Information:**

The online version contains supplementary material available at 10.1007/s00284-024-03791-w.

## Introduction

Water quality monitoring for microbial contamination is crucial for public health protection. Water is a universal solvent as well as the main ingredient in pharmaceutical products and can constitute a distinct source of microbiological contamination. In water-based environments, microorganisms, especially Gram-negative bacteria, can proliferate and develop even in the presence of extremely low amounts of nutrients. Therefore, stringent quality control should be in place to test water and liquid products used in the manufacturing of pharmaceutical products since the mere presence of water constitutes a high potential for microbial growth [1]. Non-sterile, water-based drug, and non-drug products are contaminated with opportunistic human pathogens that have resulted in product recalls within the United States (U.S.). A report surveying U.S. Food and Drug Administration (FDA) recalls from 2012 to 2019 [2] noted that *Burkholderia* spp. accounted for the greatest number of non-sterile drug recalls (105 recalls), followed by *Ralstonia pickettii* (45 recalls) and *Salmonella* spp. (28 recalls). Microbial contamination accounted for 77% of non-sterile and 87% of sterile drug recalls, indicating inadequate microbiology testing practices or faulty production practices. Most of the microbial contamination can be traced to pharmaceutical-grade water, as well as water distribution systems. Under the FDA Inspection Technical Guides (Water for Pharmaceutical Use|FDA), subject “Water for Pharmaceutical Use” non-potable water, potable water, purified water, and high-purity water are used in the manufacture of pharmaceutical products. Subsequently, water used in this process poses a serious risk of microbiological contamination of the final products, particularly in cases where proper testing procedures are not followed. The presence of certain microorganisms in non-sterile preparations has the potential to reduce or even inactivate the therapeutic activity of drug products and to adversely affect human health. The U.S. Pharmacopeia (USP) < 1111 > sets acceptance criteria for the presence of certain microorganisms in non-sterile preparations based on the route of administration [3]. Furthermore, USP < 60 >, < 61 >, < 62 >, and < 63 > tests are designed to demonstrate compliance with these requirements by quantifying the presence of specified microorganisms [4–7]. More recently, the FDA pharmaceutical microbiology manual [8] has advised manufacturers to identify specified microorganisms. Current good manufacturing practices (cGMP) require only that viable bacteria are not recovered from products; therefore, only culture-based methods (*i.e.*, for determining sterility and microbial limits) are considered cGMP. Additionally, USP has traditionally relied on the least common denominator of growth-based cultivation for testing methods [3, 9].

Historically, investigations of microbial communities have relied on culture-based methodologies. However, less than 1% of bacterial species in environmental communities are thought to be culturable on standard laboratory growth media [10]. For example, although BCC bacteria can grow and remain viable in hot or cold distilled water, most cells perish when transferred to trypticase soy broth (TSB) medium [11]. Recently, we recommended the use of oligotrophic media that allow for improved recovery of BCC organisms present in distilled water or antiseptic samples [12]. Improved detection techniques are needed to ensure pharmaceutical product quality and patient safety since BCC are capable of growing in low-nutrient conditions, resistant to antimicrobials, and have pathogenic potential [13].

Metagenomic DNA sequencing assesses the presence and the relative abundance of microorganisms, offering a more comprehensive view of the genetic complexity of natural and engineered microbial communities [14–23]. As such, this approach seems appropriate for testing for the presence of bacterial populations rather than growth-based methods to detect “specified microorganisms,” which may not be culturable. A significant benefit of this approach is the detection and identification of certain microorganisms, including BCC, in pharmaceutical manufacturing that were previously missed or not identified by culture-dependent methods. Therefore, it is important to evaluate the potential of an incorporated metagenomics approach used in water analysis of non-sterile pharmaceutical products to detect specific microorganisms.

While evaluating non-sterile pharmaceutical products, a variety of microorganisms (such as bacteria, fungi, viruses, and archaea) may be found and/or encountered, including specified microorganisms at acceptable criteria levels found in both groundwater and water-based pharmaceutical products. In the study reported here, using metagenomic sequencing, we sought to detect eight taxa, namely *Escherichia coli, Pseudomonas aeruginosa*, *Salmonella* spp.*, Staphylococcus aureus, Clostridium* spp., *Candida albicans* [5, 6], BCC [4, 9], and *Mycoplasma* spp. (USP < 63 >) [7] in “real-world” (potable and non-potable) groundwater samples from two geographical locations.

## Materials and Methods

### Experimental Scheme

**Fig. 1:**
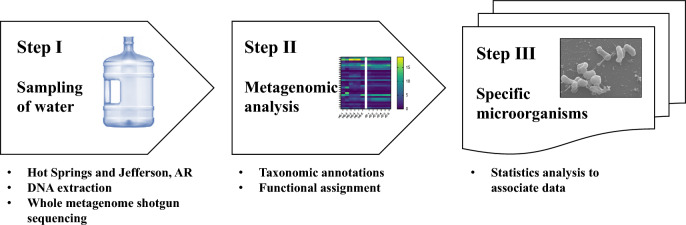
Overview of the metagenomic analysis to detect specified microorganisms in groundwater. This process is composed of three steps: Sampling, DNA extraction, and whole metagenome shotgun sequencing (Step I), metagenomic analysis, including taxonomic annotations and functional assignment (Step II), and visualization of specific microorganisms, including statistics analysis to associate data (Step III)

The experimental strategy to detect specified microorganisms in groundwater is outlined in Fig. 1. This strategy is composed of three steps,

- Step 1: Sampling, DNA extraction, and whole metagenome shotgun sequencing.
- Step 2: Metagenomic analysis, including taxonomic annotations and functional assignment, and.
- Step 3: Visualization of specific microorganisms, including statistics analysis to associate data.

### Sampling

Groundwater samples were collected from two different locations (Table 1). At Hot Springs, we collected potable water, while the non-potable water samples collected at Jefferson, came directly from cold water wells without any treatment. Using a sterilized 5-L bottle, groundwater was collected from cold water wells on six occasions in the city of Hot Springs, Arkansas (AR) (Latitude: 34°51′52.83″, Longitude: −93°04′54.258″) and on six occasions in Jefferson, AR (Latitude: 34°31′6.992″, Longitude: −93°2′57.083″) between February and August 2022. The July samples were repeatedly tested to demonstrate the reproducibility of the assay or technique. For each of these 12 collections, a total of 10 L of groundwater were filtered through 10 membrane filters (0.2 μm × 45 mm, Whatman, Thermo Fisher Scientific, MA, USA) [19].

**Table 1:** DNA concentration of the metagenomics study

Sampling site	Sampling date	Sample name	DNA conc (ng/µL)	Linear amplified DNA conc (ng/µL)	Final library DNA conc (ng/µL)	Average library size (bp)
Hot Springs, AR	2/7/2022	HS-1	7.1	43.6	39.4	664
	6/30/2022	HS-2	7.7	64.8	30	681
	7/14/2022	HS-3	3.9	58	27.6	747
	7/15/2022	HS-4	4.9	59.2	34.4	703
	7/18/2022	HS-5	3.3	63.2	18.6	752
	8/18/2022	HS-6	6.6	47.6	21.8	743
Jefferson, AR	2/15/2022	JE-1	23.1	80.4	29.6	739
	6/16/2022	JE-2	12.8	53.6	32	704
	7/5/2022	JE-3	15.3	58.8	35.2	647
	7/7/2022	JE-4	11.4	48.8	32.6	699
	7/13/2022	JE-5	11.8	50.8	37	648
	8/22/2022	JE-6	13.5	67.2	20.6	707

## DNA Extraction and Whole Metagenome Shotgun Sequencing

Ten-liter groundwater samples were filtered (1 L per filter) using 0.2 µm rated (and higher) pore size Whatman Supor 200 filters (Thermo Fisher Scientific, MA, USA). Each filter was excised, then placed into 2-mL Qiagen PowerBead tubes, and total DNA was extracted using Qiagen DNeasy UltraClean Microbial Kit (Qiagen, Germantown, MD, USA) according to the manufacturer’s instructions. The initial concentration of DNA was evaluated using a NanoDrop ND-2000 spectrophotometer (Thermo Fisher Scientific, MA, USA) and the Qubit® dsDNA HS Assay Kit (Life Technologies, Carlsbad, CA, USA). Due to low DNA concentration of the samples, linear amplification was carried out for all 12 samples using a REPLI-g Midi kit (Qiagen, Germantown, MD, USA). The linear amplified DNA was cleaned using DNEasy PowerClean Pro Cleanup Kit (Qiagen, Germantown, MD, USA) and concentrations were evaluated (Table 1) using the Qubit® dsDNA HS Assay Kit. Approx. 50 ng of DNA was used to prepare the library using Illumina DNA Prep (M) Tagmentation library preparation kit (Illumina, San Diego, CA, USA) following the manufacturer’s user guide by MR DNA (www.mrdnalab.com Shallowater, TX, USA). The samples underwent simultaneous fragmentation and addition of adapter sequences. These adapters were utilized during a limited cycle PCR in which unique indices were added to the samples. Following the library preparation, the final concentration of the libraries (Table 1) was measured using the Qubit® dsDNA HS Assay Kit, and the average library size (Table 1) was determined using the Agilent 2100 Bioanalyzer (Agilent Technologies, Santa Clara, CA, USA). The libraries were then pooled in equimolar ratios of 0.6 nM and sequenced with 150-bp paired end for 300 cycles using the NovaSeq 6000 system (Illumina, San Diego, CA, USA).

## Metagenomic Analysis

The paired end sequenced files were uploaded to the MetaGenome Rapid Annotation Subsystems Technology (MG-RAST, v3.0 (http://www.mg-rast.org) server by MR DNA (www.mrdnalab.com Shallowater, TX, USA) [24, 25]. MG-RAST is an open source, open-submission platform used to perform taxonomic and functional analyses of environmental datasets. Taxonomic annotations of the metagenome reads were performed against RefSeq databases. Subsystems under SEED were used for functional abundance analysis. All the annotations were performed using the default parameters in the MG-RAST pipeline (maximum *e* value of 10^–5^, a minimum identity of 60%, and minimum alignment length of 20 bp). Taxonomic assignment for the bacterial domain was done at phylum and genus levels. The SEED subsystem-based functional assignment was retrieved by applying filters at various hierarchical levels of classification to compute relative abundance of genes involved in different metabolic functions. For resistome profiling, the metagenomic dataset were analyzed for reads grouped at ‘Resistance to antibiotics and toxic compounds’ (level 2) of ‘Virulence, Disease, and Defense’ (level 1) of the SEED subsystems annotation. Level 3 and function-level subsystems were also analyzed for a deeper understanding of the resistome profiles.

## Visualization of Specific Microorganisms and Statistics Analysis

Based on the best taxonomic assignment for the bacterial domain, considering an *Escherichia coli, Pseudomonas aeruginosa*, *Salmonella* spp.*, Staphylococcus aureus, Clostridium* spp., *Candida albicans*, BCC, and *Mycoplasma* spp., each sequence was classified into its genus level using Microsoft Excel. Alpha-diversity metrics were calculated using Microsoft Excel [26], which included observed species, Shannon, Simpson, Pielou, and Abundance-based Coverage Estimator (ACE); the observed species index measures the number of species per sample; ACE indexes estimate species richness; Shannon and Simpson indicate species distribution diversity and evenness. A Venn diagram calculates the intersection(s) of the list of elements (https://bioinformatics.psb.ugent.be/webtools/Venn/). Pearson correlation, principal coordinate analysis (PCoA), and heatmap comparison were performed using GraphPad Prism software (v.10.0). Statistical differences in the functional annotation of level 3 subsystems were visualized using the GraphPad Prism 9 software package.

## Results

A total of 12 sequencing results were obtained from six water samples from Hot Springs and six from Jefferson, generating a total of 71,185,593 sequencing reads. After removing sequences of low-quality (percent identity < 60), 52,958,916 sequencing reads were used for bacterial diversity and community analyses (Table 2 and Supplementary Table S1) and 18,226,677 reads for functional analysis (Fig. 5 and Supplementary Table S2). In this section, we will evaluate the taxonomic composition, characterize a major metabolic pathway, and assess the presence of specified organism of the water sampled.

**Table 2:** Alpha-diversity of microbiome in 12 groundwater samples

Sample site	Sample names	Reads	Observed species	Shannon	Simpson	Pielou evenness	ACE
Hot Springs, AR	HS-1	7,193,043	1255	4.33	0.959	0.606	1254.3
	HS-2	4,529,354	1218	4.86	0.968	0.684	1217.3
	HS-3	5,285,491	917	2.68	0.877	0.394	916.6
	HS-4	1,125,772	1097	1.89	0.653	0.269	1096.3
	HS-5	1,702,124	1100	2.69	0.879	0.385	1099.4
	HS-6	1,512,284	1056	4.66	0.980	0.639	1054.3
Jefferson, AR	JE-1	4,041,585	1448	4.97	0.967	0.683	1477.2
	JE-2	4,742,945	1465	5.99	0.991	0.822	1464.3
	JE-3	4,033,241	1441	6.03	0.991	0.829	1440.3
	JE-4	5,262,548	1434	5.93	0.989	0.816	1433.2
	JE-5	4,950,006	1450	6.06	0.991	0.833	1449.3
	JE-6	8,580,523	1481	6.02	0.993	0.824	1480.3

The observed species index shows the number of reads actually observed; Shannon and Simpson indices measure biodiversity; Pielou evenness index reflects the microbial species evenness; ACE index reflects the microbial species richness

## Overview of the Metagenomic Data

In the Hot Springs groundwater samples, the average of reads was lower than that found in Jefferson groundwater samples. Specifically, the groundwater samples obtained in Hot Springs in July showed lower diversity indexes compared to the other groundwater samples collected in Hot Springs. The diversity in the groundwater microbiomes of Jefferson were similar in each sample.

**Fig. 2:**
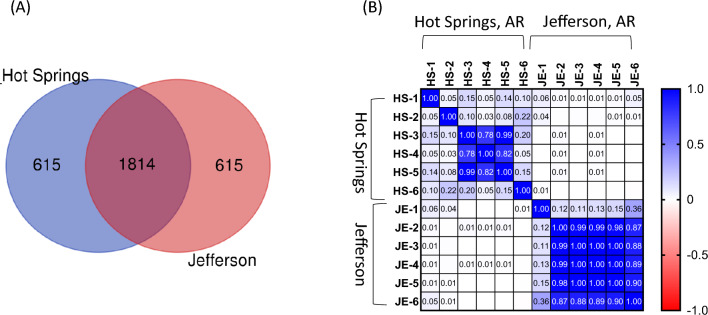
Number of reads of annotated species in cold water samples collected in the city of Hot Springs and Jefferson from February to August 2022. **a** Venn diagram of the number of reads in different groundwater samples. **b** Heatmap of Pearson correlation coefficients at the genus level

Subsequently, shared and unique reads were analyzed among groundwater samples collected in Hot Springs and Jefferson. Figure 2a shows that 1814 reads were shared among the two sampling sites. Moreover, 615 reads were uniquely found in Hot Springs and Jefferson. The complete composition of annotated reads is shown in Table S1 in Supplementary Materials. Figure 2b shows the correlation between all species (*n* = 2432) in the Hot Springs and Jefferson samples based on Pearson’s correlation analysis. In Hot Springs, the correlation coefficient of July (HS-3, HS-4, and HS-5) samples was above 0.78 (*P* > 0.05). However, coefficients with February (HS-1), June (HS-2), and August (HS-6) were lower compared to other samples. In Jefferson, the correlation coefficients of June (JE-2), July (JE-3, E-4, and JE-5) and August (JE-6) samples were above 0.87 (*P* > 0.05), whereas coefficients with February (JE-1) were lower compared to other samples.

## Taxonomic Composition of Groundwater Microbiomes

**Fig. 3:**
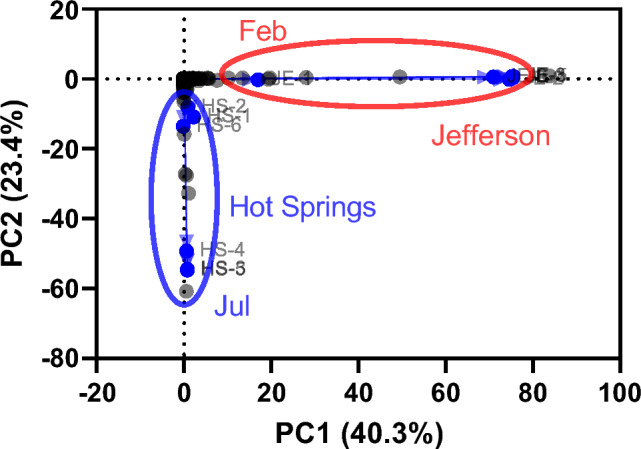
Principal coordinate analysis (PCoA) of the microbiota found in Hot Springs and Jefferson

PCoA based on parallel analysis revealed significant differences in the microbiome composition of groundwater samples collected in Hot Springs and Jefferson from February to August 2022 (Fig. 3). In PCoA, it is noteworthy that the microbiota composition of HS-1 (Feb), HS-2 (Jun), and HS-6 (Aug) in Hot Springs, as well as JE-1 (Feb) in Jefferson, exhibited a stronger correlation, in line with the Pearson correlation results. This correlation was more pronounced when compared to the microbiota composition of HS-3, HS-4, and HS-5 (Jul) in Hot Springs and JE-2 to JE-6 (Jun to Aug) in Jefferson.

The structural composition of groundwater samples was characterized at the domain level (Supplementary Fig. S1a, b). All three domains, in addition to viruses, were identified in the groundwater samples. Bacteria were dominant in HS-1 (Feb), HS-3 to HS-5 (Jul), and HS-6 (Aug) with a relative abundance of > 79%, except for HS-2 (Jun) samples collected in Hot Springs (Supplementary Fig. S1a). Similar results were also obtained with JE-1 (Feb) samples collected in Jefferson (Supplementary Fig. S1b). However, the most abundant domain in Jefferson was either bacteria (36–74%) or archaea (24–62%). Furthermore, archaea were more represented in the structure of the Jefferson samples than in the Hot Springs samples. Within Hot Springs, the HS-2 sample was characterized by a dominance of the *Ascomycota* and *Basidiomycota* phyla, with some contribution from the HS-6 sample. Among the source groundwater type, the patterns were similar for both types of groundwater samples, only archaea were slightly more abundant (0.3%) in groundwater samples. In all groups, *Proteobacteria, Bacteroidota, Firmicutes, Actinobacteria,* and *Cyanobacteria* were the main five phyla (over 95% of relative abundance) (Supplementary Fig. S1c, d). Overall, *Proteobacteria,* and *Bacteroidota* were the two most abundant phyla at both sites. However, the abundance of other phyla (*Actinobacteria,* and *Cyanobacteria*) was higher in groundwater samples HS-1 (Feb) and HS-6 (Aug) collected from Hot Springs (Supplementary Fig. S1b). In addition, the abundance of *Proteobacteria* in groundwater samples of HS-3 to HS-5 (Jul) was higher than in HS-1 (Feb) and HS-6 (Aug) in Hot Springs, whereas the relative abundance of *Proteobacteria* showed a constant trend in groundwater samples collected in Jefferson.

## Characterization of Functional Groundwater Microbiome

**Fig. 4:**
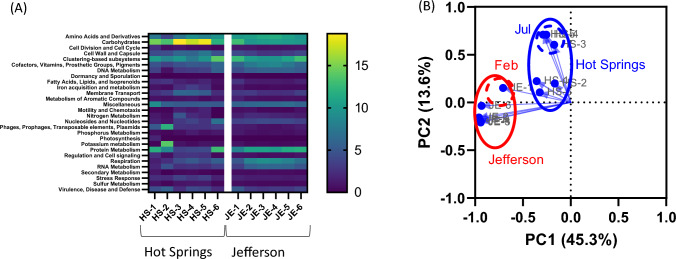
Heatmap **a** and principal coordinate analysis (PCoA) **b** of relative abundance of the 28 abundant metabolic pathways (subsystem function) in the metagenomes found in groundwater samples from Hot Springs and Jefferson, AR

Metagenome functions were predicted using GraphPad based on level 3 of a subsystem function. Figure 4a shows the heatmap of the 28 levels 1 subsystem functions of the groundwater microbiomes in samples, whereas Supplementary Table S2 summarizes level 1, level 2, level 3, as well as their respective functions. The most prevalent among these are level 1 subsystem functions, accounting for 15.7% ± 2.8% carbohydrates (ranging from 11.8 to 18.8%) and 9.9% ± 2.2% clustering-based subsystems (varying from 7.5 to 13.9%) in cold water samples collected in the city of Hot Springs. In contrast, these functions were primarily represented as 10.6% ± 1.2% and 10.9% ± 1.5%, respectively, in Jefferson groundwater samples. The 10.6% ± 0.7% amino acids and derivatives (9.7–11.6%) were also contributed groundwater samples collected in Jefferson. In contrast, the subsystem function of virulence, disease and defense was predicted in 3.8% ± 1.3% (2.3–4.5%) and 3.5% ± 0.8% (2.8–5.1%) in Hot Springs and in Jefferson groundwater samples, respectively.

Predictive subsystem functions were used to generate a PCoA plot. Water samples collected in Hot Springs and in Jefferson clustered separately (Fig. 4b). Consistently, in PCoA of the microbiome composition, HS-1 (Feb) and JE-1 (Feb) clusters were more separated from HS-3, HS-4, and HS-5 (Jul) clusters from groundwater samples collected in Hot Springs and JE-2 to JE-6 (Jun to Aug) clusters from Jefferson groundwater samples, respectively.

In ‘Resistance to toxic compounds’ (level 2) from ‘Virulence, disease, and defense’ (level 1), cobalt–zinc–cadmium resistance was predicted mainly in Hot Springs groundwater samples to be 1.1% ± 0.9% and in Jefferson groundwater samples to be 0.95% ± 0.4%, respectively (Supplementary Fig. S2). Arsenic resistance was predicted to 0.2% ± 0.3% in Hot Springs groundwater samples and 0.25% ± 0.1% in Jefferson groundwater samples, respectively. Furthermore, resistance to antibiotics (level 2), including multidrug resistance efflux pumps and BlaR1 family regulatory sensor-transducer disambiguation were observed at a comparable relative abundance in between Hot Springs (0.56% ± 0.5% and 0.42% ± 0.3%) and Jefferson (0.39% ± 0.4% and 0.67% ± 0.1%) groundwater samples. The relative abundance of beta-lactamase, erythromycin resistance, fosfomycin resistance, methicillin resistance in *Staphylococcus*, multidrug efflux pump in *Campylobacter jejuni* (CmeABC operon), multiple antibiotic resistance MAR locus, polymyxin synthetase gene cluster in *Bacillus*, resistance to fluoroquinolones, and resistance to vancomycin was relatively lower than 0.13% in both Hot Springs and Jefferson groundwater samples.

## Presence of Specific Microorganisms

We observed totals of 436–525 and 561–557 genera in groundwater samples collected from Hot Springs and Jefferson, respectively. Among these, we identified all eight of the microbial taxa we sought (*i.e., Burkholderia* spp., *Escherichia* spp.*, Pseudomonas* spp., *Salmonella* spp.*, Staphylococcus* spp.*, Clostridium* spp.*, Candida* spp.*,* and *Mycoplasma* spp.) in both Hot Springs and Jefferson. *Escherichia* spp.*, Pseudomonas* spp., and *Salmonella* spp. belong to the *Gammaproteobacteria* Class; *Burkholderia* spp. belong to the *Betaproteobacteria* Class in *Proteobacteria* phyla; *Staphylococcus* spp. and *Clostridium* spp. belong to the *Bacilli* Class and *Clostridia* Class in *Firmicutes* phyla, respectively. *Mycoplasma* spp. belong to the *Mollicutes* Class in *Mycoplasmatota* phylum. *Candida* spp. belong to the *Saccharomycetes* Class in *Ascomycota* phylum in the *Eukaryota* Domain.

**Fig. 5:**
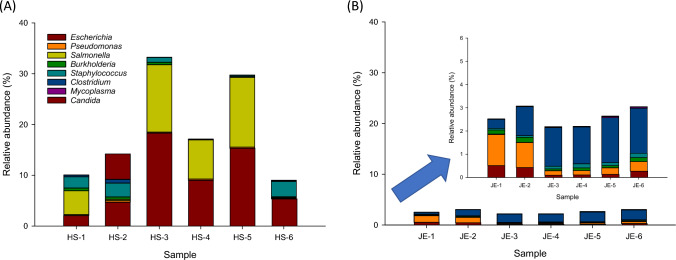
Detection of specified microorganism genera in the analyzed groundwater samples from Hot Springs a and Jefferson b. Results are expressed as relative abundance of reads

The genus-level relative abundance of specified microorganisms was observed less than 18.3% of relative sequencing read abundance in Hot Springs and Jefferson (Fig. 5). By calculating the percentage of the shared genera detected in Hot Springs versus Jefferson, we observed that *Escherichia* and *Salmonella* were mainly detected in groundwater samples collected in Hot Springs with 9.1 ± 6.4% and 6.6 ± 6.0%, relative to Jefferson (Fig. 5a). *Clostridium* and *Pseudomonas* were mainly identified in groundwater samples collected in Jefferson with 1.5 ± 0.6% and 0.6 ± 0.5%, relative to Hot Springs (Fig. 5b). In Hot Springs groundwater samples (Fig. 5a), *Staphylococcus* spp. *Candida* spp., *Burkholderia* spp., *Clostridium* spp., and *Pseudomonas* spp. were observed 1.5 ± 1.3%, 0.9 ± 2.0%, 0.3 ± 0.2%, 0.2 ± 0.3%, and 0.2 ± 0.1%, respectively. *Mycoplasma* spp. were observed very rarely at 0.004 ± 0.01%. In Jefferson, *Escherichia* spp., *Burkholderia* spp., and *Staphylococcus* spp. were observed 0.3 ± 0.2%, 0.14 ± 0.1%, and 0.12 ± 0.05%, respectively. *Salmonella* spp., *Candida* spp., and *Mycoplasma* spp. were observed less than 0.04% (Fig. 5b).

## Discussion

The concern about “specified microorganisms” in pharmaceutical products is not a new public health issue and has been addressed in the general scientific literature, including in FDA publications. Microbial examination of non-sterile pharmaceutical products is performed according to methods recommended in the texts of U.S. Pharmacopeia (USP) < 60 >, < 61 >, < 62 >, < 63 >, and < 1111 > [3–7]. The use of conventional culture-dependent methods is limited by their low sensitivity. In fact, we found that the Jefferson water samples contained less than 10^4^ CFU/mL using 1/10 Tryptic Soy Agar (1/10 TSA) and TSA, whereas no growth was observed in the Hot Springs water samples. Additionally, neither of the studied water samples yielded any growth in *Burkholderia cepacia* selective agar (BCSA; bioMérieux), indicating the limitations of the conventional approach to recovering these organisms (data not shown). However, metagenomic analysis utilizes high-throughput sequencing techniques to identify specific microorganisms and to explore how these microorganisms adapt functionally within cold drinking groundwater environments. Metagenomic analysis can provide new insights into rapid diagnostics and monitoring, which, when applied as a manufacturing process control to analyze failures in non-sterilized pharmaceutical products, may be useful in protecting public health. In that perspective, an initial study was conducted using groundwater samples from two locations between February and August 2022 in order to assess the presence of specified microorganisms using a metagenomic approach. July samples were subject to repeated measurements as technical replicates which demonstrated the reproducibility of the metagenomic assay. The genus-level relative abundance of specified microorganisms was observed less than 18.3% of relative abundance. *Escherichia, Salmonella, Clostridium,* and *Pseudomonas* were mainly detected in Hot Springs and Jefferson.

Consistent with previous work, we identified bacteria, eukaryotes, archaea, and viruses in groundwater samples [19, 23, 27]. Bacteria were the predominant group in Hot Springs groundwater, with the exception of one sample in which fungi (*Ascomycota* and *Basidiomycota*) were dominant taxa. The observation that fungi dominated just one out of six samples seems to corroborate the findings of Grabinska-Loniewska et al. [28], which suggested occasional fungal contamination of water samples. The occurrence of *Ascomycota* in groundwater samples has also been attributed to the formation and propagation of spores. These spores have a tendency to aggregate with each other and with other particles making them more resistant to water disinfectant treatment compared to bacteria [27]. In Jefferson, bacteria and archaea were predominant, which is in agreement with previous results [19]. Triplicates of groundwater samples collected in July 2022 from Hot Springs as well as Jefferson shared a similar composition, which is expected from biological replicates.

*Proteobacteria* have also been reported as the most abundant phylum in the municipal drinking water distribution system [20, 21]. Additionally, *Actinobacteria* and *Proteobacteria* comprise the majority of bacterial communities in drinking fountains, sparkling natural mineral water, non-mineral bottled water, and tap water [19]. In contrast, *Bacteroidota* and *Firmicutes* have been found to be more prevalent than *Proteobacteria* in biofilms, bulk water samples, and drinking water distribution systems [23]. *Cyanobacteria* and *Bacteroidetes* were observed to be the two major bacterial phyla during a bloom in a drinking water treatment plant [29]. In this study, *Proteobacteria* were identified as the most dominant phylum in Hot Springs and Jefferson samples. It is worth noting that while Jefferson’s non-potable water contained fewer than 4% of these specified organisms, the potable water source in Hot Springs harbors over 15% of them. Interestingly, there is a clear difference between results obtained in February 2022, June 2022, and in July 2022. Compared to July samples, those collected in February harbored mainly *Actinobacteria* and *Cyanobacteria*. It has been reported that microorganisms respond differently to environmental changes that affect the microbial community diversity and composition with substantial fluctuation throughout the four seasons [30, 31]. This may be due to seasonal differences between those two time points, with a cold and drier period occurring in February (average temperature 8.3 °C, average precipitation 104.14 mm), compared to higher average temperatures and higher rainfall in July (average temperature 27.7 °C, average precipitation 106.68 mm) (Hot Springs, Arkansas Travel Weather Averages (Weatherbase)). In contrast, such variation was not observed in Jefferson groundwater.

Carbohydrate-based level 1 subsystems showed the highest relative abundance in groundwater from Hot Springs and Jefferson. Carbohydrates are an important energy source for essential growth, metabolism, and function. Clustering-based subsystems was the second relative abundant subsystem. Clustering-based subsystems are located in the genetic regions that are collocated to functional genes in the genomes of different taxa, but their functions are not well understood [32]. Amino acids and derivatives were the third most abundant level 1 subsystem. The biogeochemical cycle of nitrogen, sulfur, and organic matter are linked to functional features of the microbiome. Therefore, further investigations of some of the chemical properties of the collected water samples remain necessary. However, virulence, disease, and defense subsystem showed a low relative abundance in Hot Springs and in Jefferson groundwater samples. The relative abundance of level 2 subsystems in virulence, disease, and defense ranged between 2.35 and 3.01% in anaerobic and aerobic sludge [33] and sediment [34]. The groundwater reservoir showed high relative abundance of this subsystem at 9% [35]. We observed a prevalence of bacterial virulence genes to be higher in Hot Springs (drinking fountain groundwater) samples compared to Jefferson samples (groundwater). Microorganisms in Hot Springs groundwater samples were more closely associated with human activity compared to Jefferson groundwater samples. Human activity affects susceptible pathogens, leading to the proliferation of genes associated with virulence, disease, and defense subsystem.

At level 3 subsystems of resistance to antibiotics and toxic compounds, all samples showed the presence of genes conferring resistance to antibiotics (*e.g*., fluoroquinolones and aminoglycosides) and heavy metals (*e.g*., cobalt–zinc–cadmium resistance). In agreement with our results, it has been found that cobalt–zinc–cadmium resistance was found in the ocean (5.7%), in mangroves (23.5%), and in terrestrial (27.5%) ecosystems [36]. A similar observation was made in the hyperalkaline Lonar Lake in India [37]. Though cobalt and zinc are essential trace elements, they can be toxic at higher concentrations, which may therefore require detoxification or removal by cation efflux pumps and perhaps by chelation. Antibiotic-resistant genes (ARG) are linked to the presence of metals [38]. Consistently, the resistome was comprised of 19 of level 3 classifications, dominated by multidrug resistance efflux pumps (46.7%) and BlaR1 family regulatory sensor-transducer disambiguation (25.2%) [39]. Multidrug resistance efflux pumps and BlaR1 family regulatory sensor-transducer disambiguation were observed at a comparable relative abundance between the Hot Springs (0.56% and 0.42%) and the Jefferson (0.39% and 0.67%) groundwater samples. This aligns with the presence of ARGs in various environments, including those existing prior to the introduction of antibiotics [39]. This may also reflect the possibility that a small fraction of the total bacterial population is resistant to antibiotics. However, further investigations are necessary to evaluate the real effect of specific bacteria on water sources.

The current USP < 1111 > has recommended microbial limits for aqueous non-sterile products of less than 100 CFU/mL of bacteria, less than 10 CFU/mL of fungi and the absence of *E. coli* in 1 g or mL of water [3]. Furthermore, non-aqueous preparations for oral use have a total aerobic microbial count limit at 10^3^ CFU/g or mL and a total combined yeasts/molds (*i.e., Aspergillus, Penicillium, Cladosporium, Fusarium, Alternaria, Mucor, Rhizopus, Trichoderma, Saccharomyces, Candida,* and others) at 10^2^ CFU/g or mL, and the absence of *E. coli* in 1 g or mL of water. As for the FDA, Title 21 of Code of Federal Regulation (CFR) §165.110 only establishes limits for coliforms (1 CFU/100 mL) and *E. coli* (absence in 100 mL) [40]. However, in most cases, a sufficient amount for detecting the fastidious bacteria by culture was 10^3^–10^4^ CFU/ml [41, 42]. Meanwhile, Brumfield et al. [19] showed that *Actinobacteria* and *Proteobacteria* are the dominant bacterial phyla detected in bottled water. Subsequently, *Betaproteobacteria*, which includes *Burkholderiales* and *Gammaproteobacteria* were the most detected microorganisms in sparkling natural mineral bottled and non-mineral bottled water, respectively. In a separate study, even though *Gammaproteobacteria* were prevalent in sparkling natural mineral bottled water, *E. coli* or enterococci were not detected in any of the drinking water samples [19]. *Proteobacteria* were consistently identified as the dominant phylum (> 60%) with 16S rRNA sequencing, respectively, in both drinking and bottled water [31, 41]. *Proteobacteria* also encompasses 85% of the isolated bacteria using the culture-based approach [43]. Additionally, *Proteobacteria* accounted for more than 99% of the reads detected with Illumina sequencing. Interestingly, fecal indicator and coliform bacteria (*E. coli* or enterococci) have been reported in private well samples and drinking water sources [44, 45]. In the current study, specified microorganisms, *i.e.*, *Gammaproteobacteria* Class *(Escherichia* spp.*, Salmonella* spp.*, Pseudomonas* spp.), *Betaproteobacteria* Class (*Burkholderia* spp.), *Bacilli* Class *(Staphylococcus* spp.), *Clostridia* Class (*Clostridium* spp.), *Candida* spp., and *Mycoplasma* spp., were detected in both sampling sites. The percentage of *Gammaproteobacteria* was greater in Hot Springs than in samples from Jefferson. The highest proportion of *Deltaproteobacteria* and *Alphaproteobacteria* was found in Jefferson. The strong presence of these specified microorganisms in both Hot Springs and Jefferson highlights the advantages of the metagenomics analysis in providing a comprehensive view of microbial population. However, it is worth noting that this approach does not discriminate bacterial OTUs that originate from viable and non-viable cells. Recently, Liu et al. [46] successfully discriminated between live and dead bacteria in human saliva and feces samples through the combination of PMAxx (an improved version of propidium monoazide [PMA]) treatment followed by DNA extraction and metagenomic analysis [46]. The PMAxx treatment, combined with our metagenomic approach, could provide better assessment to ensure effective monitoring of specified microorganisms in pharmaceutical-grade water. These results show the advantage of exploiting microbial fingerprints in assessing the quality of water used in manufacturing of non-sterile pharmaceutical products. Water being the most important raw material in pharmaceutical settings, it is recommended that it be periodically tested for the presence of bacteria in order to remain compliant with drinking water standards.

## Conclusion

Overall, this study contributes to a better understanding of the structure and function of groundwater microbiomes in Hot Springs and Jefferson, AR. Using shotgun metagenomic sequencing, genus *Escherichia* (including *Escherichia coli*) and *Salmonella* (including *Salmonella enterica*) were among the specified microorganisms that were detected mainly in Hot Springs. In Jefferson, genus *Clostridium* (including *Clostridium sporogenes*) and *Pseudomonas* (including *Pseudomonas aeruginosa*) were most often identified. The presence of specified microorganisms in groundwater is undesirable due to their pathogenic potential. Multidrug resistance efflux pumps and BlaR1 family regulatory sensor-transducer disambiguation were observed as the major resistance pathways. This study provides insight into the groundwater genetic pool and the function of the genes involved. The research also suggests practical applications for the implementation of upgraded methodologies, and seasonal (periodic) evaluations to microbial risk analysis of water sources used for pharmaceutical products. Our non-culture-based monitoring techniques were unable to distinguish live from intact dead cells in bacterial samples. Future work will aim at counting viable microbes in unprocessed as well as processed water. This will enable us to assess the levels of viable bacteria cells we might expect to see in processed water used in the production of pharmaceuticals.

### Supplementary Information

Below is the link to the electronic supplementary material.

Supplementary file1 (PPTX 175 KB)

Supplementary file2 (XLSX 277 KB)

Supplementary file3 (XLSX 724 KB)
