# Supplementary material for: A Comparative Metagenomic Analysis of Specified Microorganisms in Groundwater for Non-Sterilized Pharmaceutical Products
Source: Curr Microbiol. 2024 Jul 17;81(9):273. doi: 10.1007/s00284-024-03791-w (PMC11255085; doi:10.1007/s00284-024-03791-w)
Supplement: Supplementary file 1 — Supplementary file1 (PPTX 175 KB) [file 284_2024_3791_MOESM1_ESM.pptx]

## Slide 1
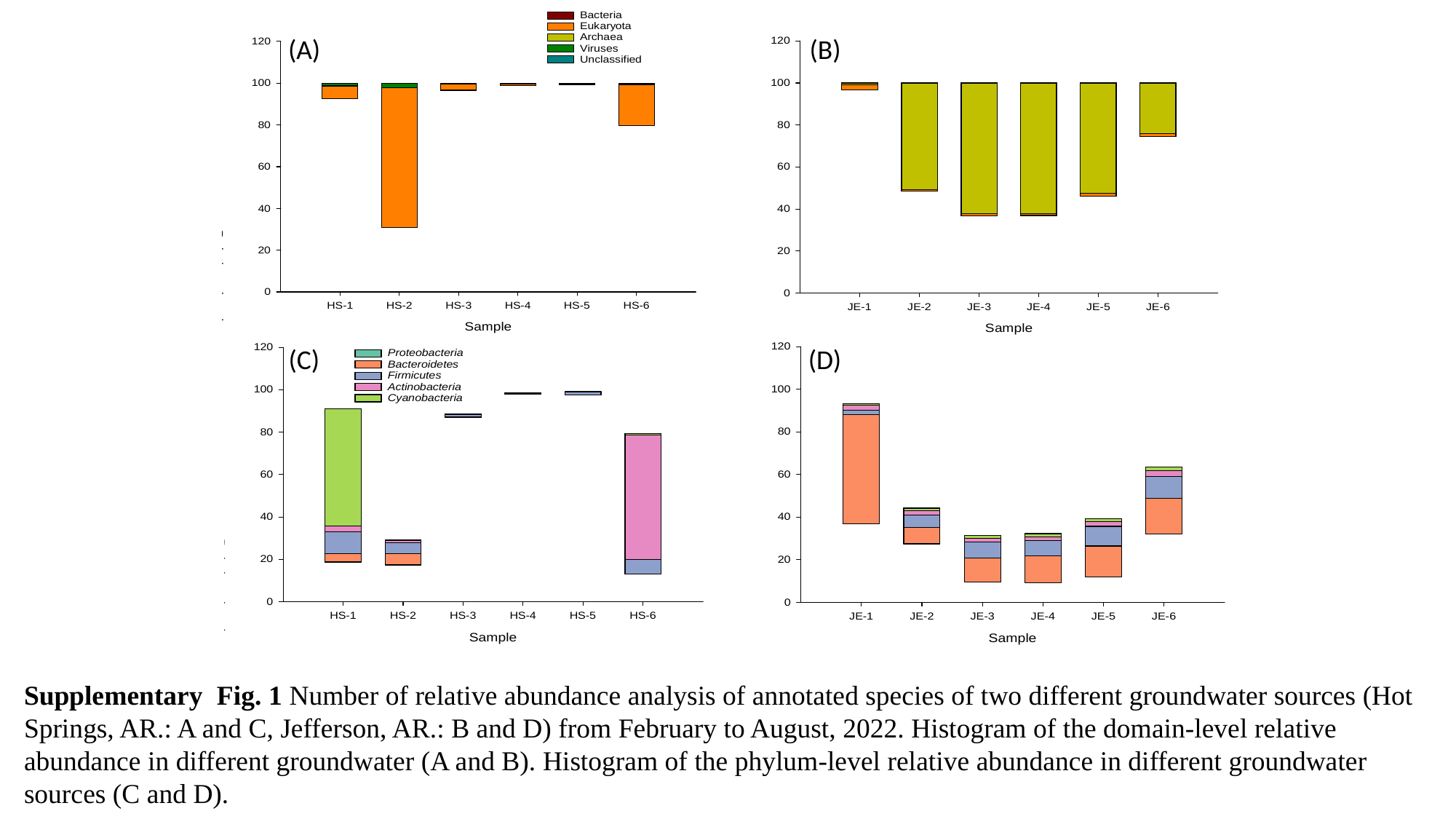

(B)
(A)
(C)
(D)
Supplementary Fig. 1 Number of relative abundance analysis of annotated species of two different groundwater sources (Hot Springs, AR.: A and C, Jefferson, AR.: B and D) from February to August, 2022. Histogram of the domain-level relative abundance in different groundwater (A and B). Histogram of the phylum-level relative abundance in different groundwater sources (C and D).

## Slide 2
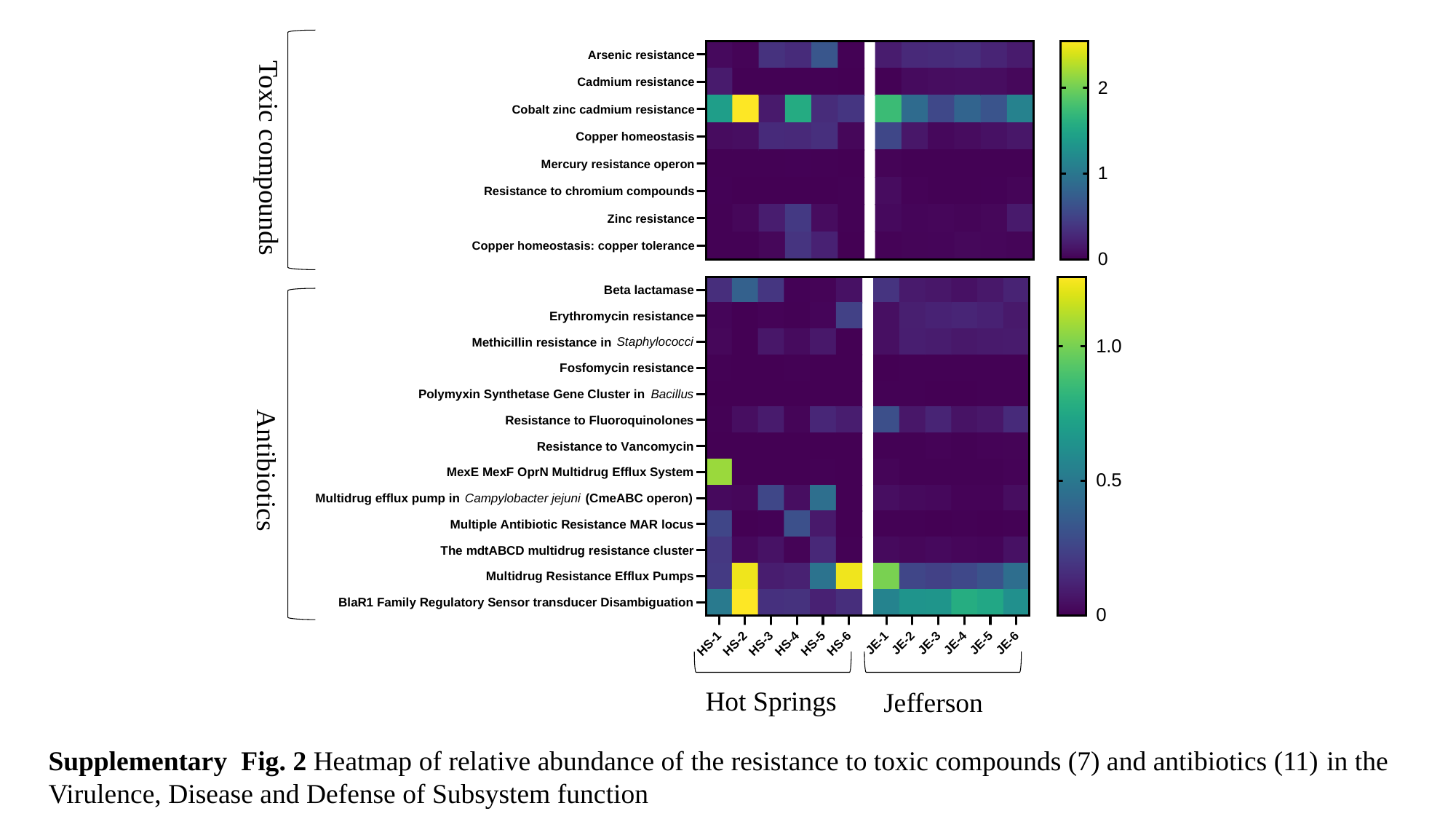

Toxic compounds
Antibiotics
Hot Springs
Jefferson
Supplementary Fig. 2 Heatmap of relative abundance of the resistance to toxic compounds (7) and antibiotics (11) in the Virulence, Disease and Defense of Subsystem function
